# Inhibition of Macrophage Functions by the C-Terminus of Murine S100A9 Is Dependent on B-1 Cells

**DOI:** 10.1155/2014/836491

**Published:** 2014-09-02

**Authors:** Rosana Lima Pagano, Natassja Foizer Moraes, Beatriz Helena De Lorenzo, Sandra Coccuzzo Sampaio, Mario Mariano, Renata Giorgi

**Affiliations:** ^1^Laboratory of Pathophysiology, Butantan Institute, Avenida Vital Brazil 1500, Butantã, 05503-000 São Paulo, SP, Brazil; ^2^Laboratory of Neuromodulation and Experimental Pain, Hospital Sírio-Libanês, Rua Coronel Nicolau dos Santos 69, Bela Vista, 01308-060 São Paulo, SP, Brazil; ^3^Discipline of Immunology, Department of Microbiology, Immunology and Parasitology, Federal University of São Paulo, Vila Clementino, 04023-900 São Paulo, SP, Brazil; ^4^Discipline of Immunology, Centro Universitário São Camilo, Ipiranga, 04263-200 São Paulo, SP, Brazil; ^5^Discipline of Immunology, Universidade Paulista, Vila Clementino, 04026-002 São Paulo, SP, Brazil

## Abstract

The protein S100A9 plays a key role in the control of inflammatory response. The C-terminus of the murine S100A9 protein (mS100A9p) downregulates the spreading and phagocytic activity of adherent peritoneal cells. Murine peritoneal cells are constituted by macrophages and B-1 cells, and the latter exert an inhibitory effect on macrophage functions by secreting interleukin- (IL-) 10. Here, we investigated the influence of B-1 cells on the inhibitory effect evoked by mS100A9p on macrophages. mS100A9p did not alter spreading and phagocytosis either by peritoneal macrophages obtained from mice deprived of B-1 cells or by bone marrow-derived macrophages (BMDM*ϕ*). Nevertheless, when BMDM*ϕ* were cocultivated by direct or indirect contact with B-1 cells treated with mS100A9p, the phagocytosis by BMDM*ϕ* was decreased, showing that the effect of mS100A9p on macrophages was modulated by B-1 cells and/or their secretory compounds. Furthermore, the inhibitory action of mS100A9p on phagocytosis by adherent peritoneal cells was abolished in cells obtained from IL-10 knockout mice. Taken together, the results show that mS100A9p has no direct inhibitory effect on macrophages; however, mS100A9p modulates B-1 cells, which in turn downregulates macrophages, at least in part, via IL-10. These data contribute to the characterization of S100A9 functions involving B-1 cells in the regulation of the inflammatory process.

## 1. Introduction

Phagocytes that express S100A8 and S100A9 proteins belong to the first group of cells that infiltrate in inflammatory sites and play a pivotal role in innate immune responses [1, 2]. These proteins have attracted a special interest due to their high cytosolic concentration in phagocytes and their high intracellular calcium-binding capacity [3, 4]. Increased plasma levels of S100A8/A9 have been found in patients suffering from a number of inflammatory disorders, including rheumatoid arthritis, inflammatory bowel disease, cystic fibrosis, psoriasis, diabetes, systemic lupus erythematosus, multiple sclerosis, and atherosclerosis, making this complex a very useful biomarker of inflammatory diseases [5–8].

Extracellular S100A9 induces neutrophil chemotaxis and adhesion [9–11], macrophage chemotaxis [12], degranulation, and activation of neutrophils [13–15] and enhances proinflammatory cytokine production by macrophages and peripheral blood mononuclear cells [16, 17]. S100A9 regulates myeloid cell function by binding to Toll-like receptors- (TLR-) 4 [13] and the receptor for advanced glycation end products (RAGE) [18] and by modulating microtubule reorganization during transendothelial migration [19], resulting in proinflammatory effects. However, S100A9 expression also has anti-inflammatory effects, by deactivation of activated peritoneal macrophages [20] and suppression of macrophage activation following phagocytosis of apoptotic neutrophils [21]. Our group has previously demonstrated that human S100A9 and the synthetic peptide corresponding to the C-terminal portion of the murine S100A9 protein (mS100A9p) have antinociceptive activity in inflammatory pain models [22–26]. Further, we showed that mS100A9p inhibits the spreading and phagocytic activity of adherent peritoneal cells stimulated or not with proteinase-activated receptor-1 [27, 28]. Thus, S100A9 has both proinflammatory and anti-inflammatory activities, emphasizing the need to further study its dual roles.

The peritoneal cavity is a unique compartment within which a variety of immune cells reside, such as different macrophages subsets [29] and B-1 cells [30]. B-1 cells represent the main B lymphocyte population in the peritoneal and pleural cavities of mice [30]. B-1 cells express high levels of IgM, low levels of IgD, and CD11b on their cell surface and can be subdivided into B-1a (CD5^+^) and B-1b (CD5^−^) cells, which develop from distinct progenitor cells [31, 32]. B-1b cells proliferate spontaneously in stationary cultures of adherent mouse peritoneal cells and differentiate into a novel type of mononuclear phagocytes [33]. B-1a cells are able to differentiate into phagocytes when cocultivated with fibroblasts [34]. Furthermore, B-1b cells leave peritoneal cavity and migrate to inflammatory sites, where they are transformed into a novel type of mononuclear phagocytes, which perform the functions of adhesion, spreading and phagocytosis [33]. The egress from the peritoneal cavity occurs by direct signals through Toll-like receptors, resulting in downregulation of integrins and CD9 expression on B-1 cells, which are essential for their mobilization and participation in immune responses [35].

B-1 cells can also influence the inflammatory milieu once they are pivotal for giant cell formation [36], wound-healing process via IL-10 [37], and inhibition of macrophage activities, also mediated by IL-10 [38]. Additionally, data support the hypothesis that B-1 cells downregulate the macrophage inflammatory response to eliminate parasites [39], exert a tolerogenic function in a model of allergic reaction [40], and modulate the innate immune system in the early phase of endotoxemia [41, 42].

Considering that peritoneal cavity is constituted by macrophages and B-1 cells, which have phagocytic activity, and that mS100A9p inhibits the activities of adherent peritoneal cells, the aim of the present study was to investigate whether the inhibitory effect induced by the C-terminus of S100A9 protein is dependent on B-1 cells and/or macrophages.

## 2. Material and Methods

### 2.1. Animals

Male BALB/c and BALB/xid mice (18–22 g) and C57BL/6 and C57BL/6 IL-10 knockout (KO) mice (25–30 g) were provided by Institutional Animal Facilities of Federal University of São Paulo (CEDEME). Male Swiss mice (18–22 g) were provided by the Central Animal House of Butantan Institute. Five animals were housed per cage, with wood shaving, at a constant ambient room with controlled temperature (22°C ± 2°C) and light/dark cycle (12/12 hours), with free access to water and mice chow pellets, at least two days before the experiments. Experimental proceedings were in accordance with the guidelines for animal experimentation, and the practices were approved by the Institutional Animal Care Committee at the Butantan Institute (CEUAIB, protocol number 072/2002).

### 2.2. Synthesis and Treatments with Peptide of S100A9

The peptide H-E-K-L-H-E-N-N-P-R-G-H-G-H-S-H-G-K-G (H^92^-G^110^; MW 2128.55 Da), which is identical to the C-terminus of mS100A9p, was synthesized based on a previously reported sequence [43]. This peptide was synthesized in solid phase by FMOC technique. The characterization and purification of the peptide were made by HPLC, and its mass was evaluated by MALDI-TOF spectrometry. The peptide was diluted in DMSO solution (15%) in Milli-Q water and stored at −20°C until the experiments. The peptide was used in the concentrations of 0.59, 1.17, and 2.35 *μ*M (0.12, 0.25, and 0.5 *μ*g, resp.), based on previously published results [27].

### 2.3. Peritoneal Cell Preparation

BALB/c, BALB/xid, C57BL/6, and C57BL/6 IL-10 KO mice were euthanized in a CO_2_ chamber and their peritoneal cavity was washed with 5 mL of cold phosphate-buffered saline (PBS), pH 7.4. After a gentle massage of the abdominal wall, the peritoneal fluid, containing resident cells, was collected. Cell viability was assessed by the Trypan blue exclusion test (>95%). Total peritoneal cells were counted in a Neubauer's chamber, and differential counts were carried out in smears stained with a panchromatic dye [44]. For all measurements, samples of individual animals were used. The assays were always performed in duplicates.

### 2.4. Bone Marrow-Derived Macrophages Culture (BMDM*ϕ*)

Bone marrow cells—freshly isolated from femora and tibia of Swiss mice, euthanized in a CO_2_ chamber—were cultured on culture dishes for 7 days in RPMI-1640 medium (Sigma, St. Louis, MO) supplemented with 20% heat-inactivated fetal bovine serum and 30% L929 cell-conditioned medium (LCCM) and used as a source of colony-stimulating factor-1 [45]. Differentiated macrophages were dispensed into dishes by incubation with PBS supplemented with 2.5 mM EDTA for 15 min. Supernatant was removed by centrifugation (1.500 rpm/5 min), and the cell pellet was resuspended with RPMI medium. Cell viability was evaluated using the Trypan blue dye exclusion method. mS100A9p was incubated for 1 h with adherent macrophages (1 × 10^5^ cells/mL) during cell spreading. Phospholipase A_2_ (PLA_2_), isolated from* Crotalus durissus terrificus* venom, which inhibits macrophage spreading and phagocytosis [46], was incubated in the concentrations of 0.85, 1.78, and 3.57 *μ*M (0.12, 0.25, and 0.5 *μ*g/mL, resp.) for 1 h with BMDM*ϕ* and used as a positive control to ensure macrophage functionality.

### 2.5. B-1 Cell Culture

B-1 cells were obtained as previously described [33, 38]. Briefly, peritoneal cells were collected from the abdominal cavity of Swiss mice by repeated lavage with 2 mL of RPMI-1640 medium. Cell viability was evaluated using the Trypan blue dye exclusion method. Cells (2 × 10^5^/mL) were dispensed on round glass coverslips (13 mm) in 24-well plates (Costar, Tokyo, Japan) and cultures incubated at 37°C in 5% CO_2_ for 40 min; after incubation, nonadherent cells were discarded. Adherent monolayers were rinsed with RPMI, and subsequently R-10 medium (RPMI-1640 containing 10% heat-inactivated fetal bovine serum) was added. Cultures were then maintained at 37°C in 5% CO_2_ for 5 days, without changing the medium, and at that time floating B-1 cells were in large numbers. Nonadherent B-1 cells were collected and submitted to cell function assays. mS100A9p was incubated for 24 h with adherent B-1 cells, during cell spreading.

### 2.6. Coculture of BMDM*ϕ* and B-1 Cells

BMDM*ϕ* (1 × 10^5^ cells) were dispensed on round glass coverslips in 24-well plates for 15 min. After this period, B-1 cells (1 × 10^5^ cells) were added directly over adherent macrophages. In another assay, B-1 cells were added to the upper compartments of transwell chambers (Costar). In both assays, in either the absence or presence of transwell chambers, mS100A9p was incubated concomitantly with B-1 cells for 1 h.

### 2.7. Spreading Assay

The spreading ability of peritoneal cells obtained from BALB/c and BALB/xid mice, BMDM*ϕ*, or B-1 cells was estimated according to a previously described method [47]. Briefly, 100 *μ*L of cell suspensions in PBS (1 × 10^5^ cells) was placed onto glass coverslips and left to adhere for 15 min at room temperature. For B-1 cells, the adherence was for 3 h. Coverslips were washed with PBS and incubated in RPMI medium at 37°C for 1 h, in the presence of mS100A9p, during cell spreading. For B-1 cells, R-10 medium was used for 24 h, incubated with mS100A9p. Cells were fixed in a 2.5% glutaraldehyde solution and the index of cell spreading was determined by phase contrast microscopy. This index was defined as percentage of spread cells in 100 cells counted. Spread cells were defined as adherent cells which changed their rounded shape to a flattened shape, showing a lower refractilebody and a higher diameter compared to unspread cells [47].

### 2.8. Phagocytic Activity

Coverslips containing adherent and spread cells of the peritoneal cavity of BALB/c, BALB/xid, C57BL/6, and C57BL/6 IL-10 KO mice or BMDM*ϕ* or B-1 cells were incubated with 1 mL of RPMI medium containing nonopsonized* Candida albicans* in an atmosphere containing 5% CO_2_ for 1 h at 37°C. The percentage of cells that phagocytosed more than three particles was determined in smears stained with a panchromatic dye [44], by examination under light microscopy.* Candida albicans* (ATCC Y-537) was cultured in 8% Sabouraud's dextrose broth (Microbiology and Mycology Laboratories, Department of Clinical Analyses, College of Pharmaceutics Science, University of São Paulo) at 30°C, for one day. Fungi were suspended in 3 mL of Dulbecco's PBS for determining* Candida albicans* count in Neubauer's chamber, and then the particles were suspended in RPMI medium for the phagocytosis assay.* Candida albicans* viability was determined by exclusion of 0.01% methylene blue (>98%). The particle count was approximately 1 × 10^6^ per coverslip.

### 2.9. Statistical Analysis

Results are expressed as means ± standard deviation (SD). Comparisons between experimental and control groups were initially tested by analysis of variance (ANOVA). The alpha level (significance level related to the probability of rejecting a true hypothesis) was set at *P* ≤ 0.05. Significant differences were then compared using Tukey's test.

## 3. Results

### 3.1. mS100A9p Did Not Alter the Spreading and the Phagocytic Activity of Macrophages Obtained from BALB/xid Mice

**Figure 1:**
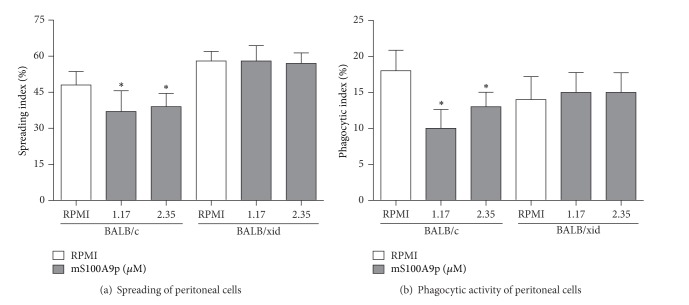
Spreading and phagocytosis assays by peritoneal cells obtained from BALB/c and BALB/xid mice after incubation with mS100A9p. (a) Peritoneal cells were adhered to coverslips (1 × 10^5^ cells/well) and incubated with mS100A9p (1.17 or 2.35 *μ*M in RPMI-1640 medium), for 1 h at 37°C in 5% CO_2_. Control adherent cells were incubated only with culture medium. (b) After spreading, cells were incubated with 1 × 10^6^ *Candida albicans* particles for 1 h. Results are expressed as means ± SD, using six animals per group. *P ≤ 0.05, when compared to the respective control group (RPMI).

In order to evaluate the involvement of B-1 cells in mS100A9p-induced inhibitory effect on macrophage spreading, we used peritoneal cells obtained from BALB/xid mice, which are deficient in B-1 cells [48]. For comparison, peritoneal cells obtained from isogenic BALB/c mice were used. mS100A9p downregulated spreading of peritoneal cells obtained from BALB/c mice in the concentrations of 1.17 and 2.35 *μ*M (23 and 19% of inhibition, resp.), when compared with control cells, incubated only with culture medium (Figure 1(a)). On the other hand, mS100A9p, in both concentrations, did not change spreading of peritoneal cells obtained from BALB/xid mice (Figure 1(a)). In relation to the phagocytic activity of* Candida albicans *particles, mS100A9p did not change the phagocytosis by peritoneal cells obtained from BALB/xid mice either (Figure 1(b)). mS100A9p inhibited the phagocytosis by peritoneal cells obtained from BALB/c mice, in the concentrations of 1.17 and 2.35 *μ*M (44 and 28% of inhibition, resp.) (Figure 1(b)).

### 3.2. mS100A9p Did Not Change the Spreading and the Phagocytic Activity of BMDM*ϕ*

**Figure 2:**
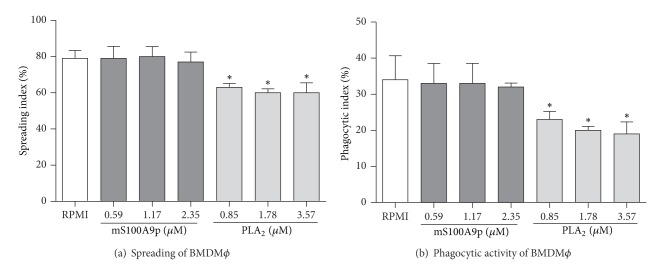
Spreading and phagocytosis assays by bone marrow-derived macrophages (BMDM*ϕ*) after incubation with mS100A9p. (a) BMDM*ϕ* adhered to coverslips (1 × 10^5^ cells/well) were incubated with mS100A9p (0.59; 1.17; or 2.35 *μ*M in RPMI-1640 medium), for 1 h at 37°C in 5% CO_2_. Phospholipase A_2_ (PLA_2_: 0.85; 1.78; 3.57 *μ*M), in the same conditions of peptide, was used as positive control. BMDM*ϕ* controls were incubated only with culture medium. (b) After spreading, BMDM*ϕ* were incubated with 1 × 10^6^ *Candida albicans* particles for 1 h. Results are expressed as means ± SD, using five samples per group. *P ≤ 0.05, when compared to the control group (RPMI).

To confirm that mS100A9p was unable to downregulate macrophage functions in the absence of B-1 cells, mS100A9p was incubated with BMDM*ϕ* during the spreading assay. mS100A9p, in concentrations of 0.59, 1.17, and 2.35 *μ*M, did not alter BMDM*ϕ* spreading (Figure 2(a)). PLA_2_, the positive control of inhibition of macrophage spreading and phagocytosis, downregulated spreading of BMDM*ϕ* (20% to 0.85 *μ*M, 24% to 1.78 *μ*M, and 24% to 3.57 *μ*M) (Figure 2(a)). Different concentrations of mS100A9p incubated for 1 h with BMDM*ϕ* during spreading did not downregulate phagocytosis of* Candida albicans* particles (Figure 2(b)). On the other hand, PLA_2_ inhibited phagocytosis by BMDM*ϕ* in all concentrations used (32% to 0.85 *μ*M, 41% to 1.78 *μ*M, and 44% to 3.57 *μ*M) (Figure 2(b)).

### 3.3. mS100A9p Downregulated Phagocytosis by BMDM*ϕ* Cocultivated with B-1 Cells

**Figure 3:**
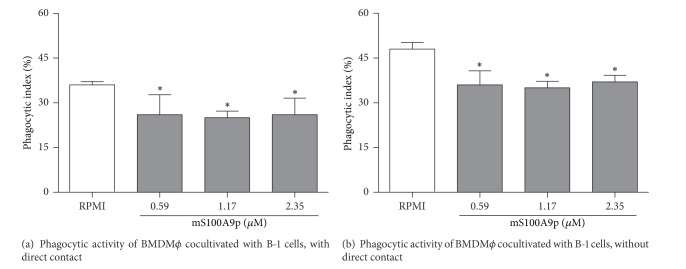
Phagocytosis assays by bone marrow-derived macrophages (BMDM*ϕ*), cocultivated with B-1 cells, treated with mS100A9p. BMDM*ϕ* adhered to coverslips (1 × 10^5^ cells/well) were incubated in the presence of B-1 cells (1 × 10^5^ cells/well) concomitant with mS100A9p (0.59; 1.17; or 2.35 *μ*M in RPMI-1640 medium), for 1 h at 37°C in 5% CO_2_. Coculture assays were evaluated with (a) or without (b) direct contact of these two cell populations, using transwell chambers. Experimental controls were cocultures of BMDM*ϕ* and B-1 cells incubated only with culture medium. After 1 h, both cocultures were incubated with 1 × 10^6^ *Candida albicans* particles for 1 h. Results are expressed as means ± SD, using five samples per group. *P ≤ 0.05, when compared to the control group (RPMI).

In an attempt to evaluate if the presence of B-1 cells was primordial to mS100A9p-induced inhibition of macrophage spreading and phagocytosis, we investigated the effect of mS100A9p on BMDM*ϕ* cocultivated directly with B-1 cells. In the presence of the B-1 cells, phagocytosis of* Candida albicans *particles by BMDM*ϕ* was inhibited 28, 31, and 28%, which corresponded to concentrations of 0.59, 1.17, and 2.35 *μ*M mS100A9p, respectively (Figure 3(a)). To verify whether the contact between these two cell types was necessary to the inhibitory effect of mS100A9p, cocultures of BMDM*ϕ* and B-1, in the presence of mS100A9p, were performed using transwell chambers. The absence of direct contact between BMDM*ϕ* and B-1 cells maintained the downregulation of the macrophage phagocytic activity in response to mS100A9p. The fall in phagocytosis was 25, 27, and 23%, corresponding to concentrations of 0.59, 1.17, and 2.35 *μ*M mS100A9p, respectively (Figure 3(b)).

### 3.4. mS100A9p Inhibited Spreading and Phagocytosis by B-1 Cells

**Figure 4:**
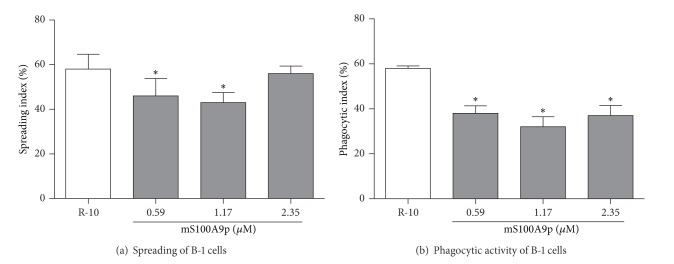
Spreading and phagocytosis assays by B-1 cells after incubation with mS100A9p. (a) B-1 cells, isolated by stationary culture of peritoneal cells obtained from Swiss mice, were adhered per 3 h to coverslips (1 × 10^5^ cells/well) and incubated with mS100A9p (0.59; 1.17; or 2.35 *μ*M in R-10, RPMI-1640 medium containing 10% of heat-inactivated fetal bovine serum, R-10), for 24 h at 37°C in 5% CO_2_. Control adherent cells were incubated only with R-10. (b) After spreading, cells were incubated with 1 × 10^6^ *Candida albicans* particles for 1 h. Results are expressed as means ± SD, using six animals per group. *P ≤ 0.05, when compared to the control group (R-10).

Considering the inhibitory effect of mS100A9p in the coculture of BMDM*ϕ* and B-1, we evaluated whether the peptide also exerted a direct action on B-1 cells. mS100A9p induced a statistically significant reduction in spreading of B-1 cells in the concentrations of 0.59 and 1.17 *μ*M (21 and 26% of  inhibition, resp.) (Figure 4(a)). In addition, mS100A9p inhibited phagocytosis by B-1 cells, at the concentrations of 0.59, 1.17, and 2.25 *μ*M (34, 45, and 36%, resp.) (Figure 4(b)).

### 3.5. mS100A9p Did Not Alter Phagocytosis by Adherent Peritoneal Cells Obtained from IL-10 KO Mice

**Figure 5:**
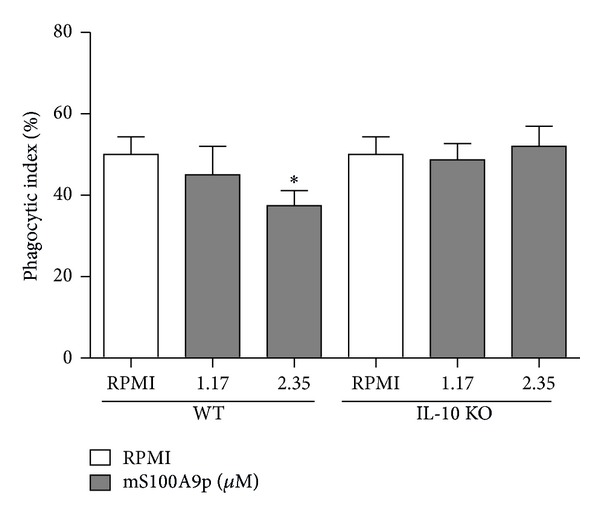
Phagocytic activity by peritoneal cells obtained from C57BL/6 wild-type (WT) and C57BL/6 IL-10 KO (IL-10 KO) mice after incubation with mS100A9p. Peritoneal cells obtained were adhered to coverslips (1 × 10^5^ cells/well) and incubated with mS100A9p (1.17 or 2.35 *μ*M in RPMI-1640 medium) for 1 h at 37°C in 5% CO_2_. Control adherent cells were incubated only with culture medium. After this time, cells were incubated with 1 × 10^6^ *Candida albicans* particles for 1 h. Results are expressed as means ± SD, using six animals per group. *P ≤ 0.05, when compared to the control group (RPMI).

Since B-1 cells secrete IL-10 [49], which inhibits murine macrophage phagocytosis* in vitro* [38], we evaluated the involvement of IL-10 in mS100A9p-induced inhibitory effect on phagocytosis by peritoneal cells obtained from IL-10 KO mice. Peritoneal cells obtained from wild-type mice (C57BL/6) were used as control. mS100A9p downregulated phagocytosis by peritoneal cells obtained from wild-type C57BL/6, in the concentration of 2.35 *μ*M (25% of inhibition), when compared with control cells incubated only with culture medium (Figure 5). The peptide, in both concentrations (1.17 and 2.35 *μ*M), did not modify phagocytosis by peritoneal cells from C57BL/6 IL-10 KO mice, when compared with control cells (Figure 5).

## 4. Discussion

S100A8/A9 are important proteins expressed by phagocytes during the inflammatory response [5]. The expression of these proteins is restricted to cells of the monocytic/granulocytic lineage, but under certain conditions they may also be expressed by keratinocytes [50, 51]. It was also demonstrated that newly arrived inflammatory macrophages, but not the resident ones, express S100A8/A9 at inflammatory sites for a short time [50, 52]. Both proinflammatory and anti-inflammatory functions have been reported for S100A8/A9, which may depend on several factors, including their concentration, receptors involved in their recognition, their posttranslational modifications, cell types studied, and mediators of the local milieu [53, 54]. Previous studies of our group demonstrated an inhibitory effect of either S100A9 or its C-terminal portion on an inflammatory nociception model [22, 24, 26], suggesting that mS100A9p induces a similar effect to that evoked by the complete protein. This evidence supports the hypothesis that this peptide has a crucial role in inflammatory events. In addition, mS100A9p downregulated the spreading and phagocytosis by adherent peritoneal cells* in vivo *and* ex vivo *[27, 28]. Considering the inhibitory effect of mS100A9p on the activities of peritoneal cells and that B-1 cells represent the main B cell population in normal peritoneal cavity of mice [55], in this study it was investigated whether the inhibitory effect induced by C-terminus of S100A9 protein is related to an action on macrophages and/or B-1.

Initially, we investigated the effect of mS100A9p on spreading and phagocytosis by peritoneal cells from BALB/xid mice, which are deficient in B-1 cells [48]. Our data showed that mS100A9p did not inhibit the functions of cells obtained from BALB/xid mice, suggesting an important role of B-1 cells in mS100A9p-induced inhibitory effect on adherent peritoneal cells. These data were supported by other studies showing that absence of B-1 cells in BALB/xid mice interfered with the inflammatory response, including the activity and proliferation of peritoneal macrophages [38, 42], control of infection [39, 56], and wound-healing process [37].

To verify that mS100A9p did not act directly on macrophage activity, the effect of mS100A9p on the functional response of BMDM*ϕ* was evaluated. Once mS100A9p did not alter the spreading and phagocytosis of BMDM*ϕ*, these data confirm that mS100A9p did not have a direct action on macrophages. PLA_2_, used as a positive control, inhibited spreading and phagocytosis by macrophages, as described elsewhere [46]. These results demonstrated that macrophages used in our assays were functioning normally. Despite the findings obtained here, suggesting that the inhibitory effect of mS100A9p is not due to a direct action on macrophages, it has been showed that mS100A9p downregulates the phagocytic activity of apoptotic neutrophils by macrophages [21]. Nevertheless, isolated macrophages have not been used, and the mS100A9p effect was observed on adherent peritoneal cells, which comprise macrophages and B-1 cells. This fact could explain the discrepancy between the results presented here and those reported by De Lorenzo et al. [21].

Using coculture assays, it is well established that the function of macrophages is largely regulated by intercellular communication, which influences the development and maintenance of the inflammatory response [57, 58]. Thus, by coculturing B-1 cells with macrophages obtained from BALB/xid mice, IL-10 secreted by B-1 cells has been demonstrated to modulate murine macrophage phagocytosis [38]. Considering these data, it was investigated whether B-1 cells treated with mS100A9p could modulate macrophage phagocytosis. In addition, in an attempt to show whether the effect of mS100A9p depended on a direct contact between B-1 cells and macrophages, the coculture of these two cell types was performed in the absence or presence of transwell inserts. Once a downregulatory effect of mS100A9p was observed in both assays, we can suggest that the inhibitory effect of mS100A9p on the adherent peritoneal cells, as previously observed [27], is mediated by B-1 cells. These results also showed that the direct contact between macrophages and B-1 cells is not essential to mS100A9p activity but is related to the secretory activity of B-1 cells.

Considering that B-1 cells differentiate into a macrophage-like cells, exhibiting the ability to phagocytose either in vitro or* in vivo* [33, 59], we decided to evaluate whether mS100A9p had an effect on B-1 cell functions. The data presented here show that mS100A9p downregulated the spreading and phagocytosis by B-1 cells, suggesting that the C-terminus of S100A9 not only modulates the action of B-1 cells, which in turn inhibits the phagocytic activity of macrophages, but also has a direct effect on B-1 cells, probably by a communication via soluble autocrine and paracrine signals. Also, we did not observe a concentration-dependent effect of mS100A9p on spreading and phagocytosis assays carried out.

Many factors have a regulatory role in macrophage activity, acting as key orchestrators of the inflammatory response. In this sense, IL-10 has been found to downregulate a number of different macrophage functions, including cytokine production and the respiratory burst [60, 61], acting thereby as an important negative regulator of cell-mediated immunity [62]. Peritoneal B-1 cells are the major producers of IL-10 and they use it to regulate their own development and/or the function of other immunocompetent cells [49, 63]. As mentioned earlier, IL-10 secreted by B-1 cells downregulates the phagocytic activity of macrophages [38] and modulates the kinetics of wound-healing process [37]. Further, B-1 cells from IL-10 KO mice were not able to inhibit macrophage functions [38].

To investigate the IL-10 participation in mS100A9p-induced inhibitory effect, we evaluated whether mS100A9p altered the phagocytic activity of peritoneal cells obtained from IL-10 KO mice. Considering that mS100A9p did not change the activity of these cells, it is plausible to suggest that mS100A9p induces the secretion of IL-10 by B-1 cells and consequently inhibits its own functions and the activities of peritoneal macrophages. In this regard, it was demonstrated,* in vitro* and* in vivo*, that B-1 cells induce macrophage polarization to an M2-like phenotype, which is anti-inflammatory and immunosuppressive in nature, evidencing that IL-10 is crucial to modulate macrophage function [64].

The mechanism by which mS100A9p regulates IL-10 secretion by B-1 cells is not known. However, both B-1 cell activation and IL-10 release are regulated by TLR-4 [65–67], and S100A9 protein acts as an endogenous TLR-4 ligand, regulating thereby myeloid cell function [13, 68, 69]. In this sense, it is plausible to hypothesize that the C-terminus of S100A9 stimulates IL-10 secretion by B-1 cells via TLR-4, suppressing thereby macrophage activities. These data emphasize the importance of S100A9 and B-1 cells to orchestrate inflammatory response and its resolution.

## 5. Conclusion

The downregulatory effects of mS100A9p on macrophages are mediated by B-1 cells, possibly via IL-10 secretion. Taken together, our data showed that mS100A9p is a good research tool to understand S100A9 function on the development and maintenance of the inflammatory process and demonstrated a pivotal role of B-1 cells mediating the action of the C-terminus of S100A9 on control of innate immunity.
